# A Mixed Methods Comparison of Urban and Rural Retail Corner Stores

**DOI:** 10.3934/publichealth.2015.3.554

**Published:** 2015-08-26

**Authors:** Jared T McGuirt, Stephanie B. Jilcott Pitts, Alice Ammerman, Michael Prelip, Kathryn Hillstrom, Rosa Elena Garcia, William J. McCarthy

**Affiliations:** 1 Department of Nutrition, Gillings School of Global Public Health, UNC Center for Health Promotion and Disease Prevention, University of North Carolina-Chapel Hill, 2200 McGavran-Greenberg Hall, Chapel Hill NC; 2 Department of Public Health, East Carolina University, Lakeside Annex 8, Room 126, Greenville NC; 3 UCLA Fielding School of Public Health, University of California, Los Angeles, CA; 4 Department of Kinesiology & Nutritional Science, California State University, Los Angeles, CA

**Keywords:** food environment, corner stores, rural, urban, spatial regression

## Abstract

Efforts to transform corner stores to better meet community dietary needs have mostly occurred in urban areas but are also needed in rural areas. Given important contextual differences between urban and rural areas, it is important to increase our understanding of the elements that might translate successfully to similar interventions involving stores in more rural areas. Thus, an in-depth examination and comparison of corner stores in each setting is needed. A mixed methods approach, including windshield tours, spatial visualization with analysis of frequency distribution, and spatial regression techniques were used to compare a rural North Carolina and large urban (Los Angeles) food environment. Important similarities and differences were seen between the two settings in regards to food environment context, spatial distribution of stores, food products available, and the factors predicting corner store density. Urban stores were more likely to have fresh fruits (Pearson chi2 = 27.0423; *p* < 0.001) and vegetables (Pearson chi2 = 27.0423; *p* < 0.001). In the urban setting, corner stores in high income areas were more likely to have fresh fruit (Pearson chi2 = 6.00; *p* = 0.014), while in the rural setting, there was no difference between high and low income area in terms of fresh fruit availability. For the urban area, total population, no vehicle and Hispanic population were significantly positively associated (*p* < 0.05), and median household income (*p* < 0.001) and Percent Minority (*p* < 0.05) were significantly negatively associated with corner store count. For the rural area, total population (*p* < 0.05) and supermarket count were positively associated (*p* < 0.001), and median household income negatively associated (*P* < 0.001), with corner store count. Translational efforts should be informed by these findings, which might influence the success of future interventions and policies in both rural and urban contexts.

## 1. Introduction

Residents of low income urban and rural areas disproportionately suffer from diet-related morbidity and mortality [1]–[6]. While there are undoubtedly many reasons for these disparities, one important cause is the unique food environment exposures experienced by both of these populations [7]. These disparities in health may result from these areas being classified as food deserts, that is food environments lacking health-promoting foods, particularly areas composed of predominantly lower income neighborhoods and communities [8], or classified as "food swamps”, that is, environments inundated with hyper-processed, energy-dense, nutrient-poor foods [9]. The common thread is that more nutrient-dense foods are typically less available to residents of both of these areas [10],[11]. A review of the literature by Larson, Story, and Nelson (2009) found that this is particularly true in low income, minority, and rural areas, which are most often burdened with poor access to more healthful foods [12]. Inadequate access is associated with reduced consumption of healthier foods like fresh, minimally processed fruits and vegetables among residents in both areas [13],[14]. In addition to less access to healthy foods, lower-income, minority, and rural neighborhoods may have greater access to energy-dense foods, particularly with a greater presence of convenience stores, which may lead to less healthy diets and higher levels of obesity [12]. Given these findings, Larson, Story, and Nelson (2009) called for a push for policy action and intervention to ensure equitable access to healthy foods [12].

Several strategies have been promoted [15] to improve the food environment, with mixed success [16]. These approaches have focused on the effectiveness of creating new food-related opportunities [17]–[19], or transforming existing opportunities in order to make healthier foods more accessible [20],[21]. Food stores have the potential to influence point of purchase decision-making regarding household food purchases, and may have sustainable long term impact by changing local demand to encourage continued availability of healthy foods [22]. Most of this work has occurred in supermarkets, where efforts have shown some promise [23]. Convenience stores, or “corner stores”, are existing venues that have received increased attention as having great potential to improve the food environment [14],[15],[24],[25]. Transforming convenience stores to better meet community dietary needs is a logical strategy for multiple reasons, including their often less healthy food offerings [24]–[31], their common presence in both limited resource urban and rural locations [25]–[29],[32]–[37], the fact that in some areas of limited food access primary food shoppers [8],[38] and children often shop at corner stores for food [39],[40], and the fact that proximity to corner stores is associated with higher obesity among youth [41].

Most of the corner store transformation work to date has focused on convenience stores, corner stores, and bodegas in urban areas, with some promising successes. In a systematic review, Gittlesohn et al (2012) [37] found that there were significant increases in availability of healthy foods, and in sales of promoted healthy foods (in all six trials that collected sales data). Only 5 of 16 (31%) studies were conducted in remote or rural areas, and most of those were in special populations and contexts, such as Native American reservations and the Marshall Islands.

Given the success of these interventions in urban areas, recent efforts have pushed for the translation of this work into more rural areas, where convenience stores, particularly in “crossroads” areas (a small rural community situated at an intersection of two or more roads), play a pivotal role in providing access to food in remote rural locations [42],[43]. To effectively translate previous corner store work, a better understanding is needed of the similarities and differences between urban and rural corner stores. In both rural and urban food deserts, where corner stores often are the dominant food store available, there are often higher rates of poverty, greater concentrations of minority populations, and lower vehicle access rates [44]. In both locations, research has indicated that more corner stores were associated with poorer health outcomes, including adjusted mortality, diabetes, and obesity rates [45].

There are also important demographic, spatial, cultural, and structural differences between urban and rural areas, which have led to distinct differences in the food environment in each area. Rural areas tend to be more sparsely populated, have lower housing values, and residents are more likely to live below poverty and have lower educational obtainment compared to urban areas, characteristics that influence business decisions about the location and types of food stores available [27],[46]. The isolation of rural households from important services and opportunities such as schools, social interactions, and work opportunities have led to disproportionately high persistent poverty in rural areas [47]. This isolation also makes access to transportation a particularly prominent factor in determining whether food is easily accessible[47]. Deller, Canto, and Brown (2015), through a multi-faceted analysis of the relationship between food access, poverty, and public health, demonstrated that this relationship is more complex in rural areas compared to urban areas, thus inferences drawn from the urban literature may not directly translate to the rural setting [47].

Some work has been done to understand the characteristics of corner stores in rural areas [42],[43],[48],[49]. Morton and Smith (2009) [49] identified unique factors related to perceptions of food access in Midwestern US rural areas using focus groups, including personal, cultural, and structural themes that influenced food access compared to urban areas. Participants identified several perceptions of differences between themselves and urban dwellers, including less access to food stores, affordable foods and food assistance benefits. Other research has indicated other issues such as limited public transportation and increased isolation as issues unique to the rural food environment [50]. Thus, these and other contextual differences might influence the success of a corner store transformation in a rural versus urban area.

There has been little direct comparison of corner stores in urban versus rural food environments, especially in terms of how they are distributed spatially and what kinds of healthy food they typically stock. Hendrickson et al (2006) [51] conducted a comparison of rural versus urban food stores (not exclusively corner stores) including food store audits, focus groups, and a survey of residents. They found that prices for foods were higher in urban compared to rural food stores (though only 5 fruit and vegetable items were assessed), though no clear examination of availability, nor differences in geographic access were assessed in the research.

There is a need to better understand if potential differences in rural versus urban areas have influenced the current presence and offerings of corner stores, which could also indicate the success of future store transformation and policy efforts. Completing in-store observations is an underutilized method that is also needed to help better clarify differences in residential access to healthy food.^12^ Furthermore, increasing our understanding of the predictors of corner store presence in rural versus urban communities can increase our understanding of the factors that create demand for corner stores in each area. The majority of this research has focused on urban food deserts, with little research on the market realities that drive retail site selection in rural areas [52],[53] Only one study [54] was found that attempted a spatial examination of access to convenience stores in a rural area, finding low presence in sparsely populated areas between towns, but they did not examine contextual characteristics (income, transportation, etc.) that might be influencing these locations. Thus, more research is needed. Additionally, there are a limited number of studies [33],[34],[55],[56] which have examined access to food stores within the same geographic area, particularly in terms of differing economic and social characteristics, in order to assess neighborhood disparities in access [12]. Sharma (2014) [57] suggested that by understanding geographic differences and recognizing spatial patterns, improvements in population health can be made through effective targeting of at risk groups with health policy approaches. This approach suggests that if there is access and food availability issues identified in particular geographic areas amongst particular subgroups, corner store transformations and healthy food access initiatives can be targeted to those areas. Thus, there is a need to examine local variation given these characteristics within each rural and urban area to see if these differences exist.

Therefore the purpose of this study was to: 1) Examine and compare contextual factors in the food environments in Lenoir County, North Carolina (NC), and Los Angeles County, California (CA), 2) Examine and compare corner store food availability through structured food store audits (Lenoir: 28 stores; Los Angeles: 161 stores), 3) Examine the distribution of stores and food products through a spatial evaluation using Geographic Information System (GIS) software, and 4) Understand the predictors of urban versus rural corner store presence by conducting a spatial regression analysis in a seven county area in rural eastern NC (Lenoir, Greene, Onslow, Craven, Duplin, Jones, and Wayne counties). Place based metrics of food accessibility, including the spatial distribution and quality of goods and services available to consumers at a given location [58], will be the focus of this study. This approach measures the food environment in aggregate, with the goal to facilitate accessibility comparisons across places [58].

## 2. Methods

The settings for this study included rural eastern North Carolina and the greater Los Angeles Urbanized area, with a focus on low income East Los Angeles. Both of these areas feature National Institutes of Health (NIH) Center for Population Health and Health Disparities (CPHHD) projects, which include ancillary projects to improve the local food environment. Given the similarity in ancillary project goals, and the relative success of the University of California Los Angeles CPHHD ongoing Corner Store Makeover Project in limited resource East Los Angeles [59], this project attempted to increase understanding of the factors that might influence the success of a similar intervention in rural eastern North Carolina. Rural eastern North Carolina is a heavily agricultural and coastal area, with some of the worst county-level health indicators in the United States [60]. Of focus for this study is Lenoir County, a county with poorer diet-related health outcomes than the rest of the state of North Carolina, and is located in the heart of the stroke belt [61]. At 10 million residents, Los Angeles County is the most populous county in the U.S [62]. A comparison of the two counties can be found in Table 1[60],[62],[63].

**Table 1.** Comparison of Lenoir (rural) and Los Angeles Counties (urban) [60],[62],[63].

Comparison Table		
	Lenoir County	Los Angeles County
Population, 2013	58,914	10,017,068
Population Density, 2010	150 per square mile	7,544.6 per square mile
Percent Latino	7.1%	48.3%
Percent Black	40.9%	9.2%
Median Household Income	$34, 440	$56,241
Children in poverty	37%	27%
Percent Obese (Adults)	34%	21%
Poor or Fair Health	25%	22%
Pct. of Total Low Income Population, Living Over 1 Mile From a Food Store	23%	1.6%
Grocery Stores, Rate (per 100,000 Pop.), 2009	30.1	20.6
Convenience Stores With No Gas, per 100,000 Pop., 2007	75.9	18.2

For this study, corner stores were defined as small grocery stores and convenience stores (by matching North American Industrial Classification System (NAICS) codes and less than $2.5 million sales). Corner stores and supermarkets (large regional and national retail chains) were identified using the Reference USA online business database during the spring of 2014, using only verified store listings. To characterize the food environment context around the corner stores, we evaluated the distance between each corner store and the nearest supermarket. Data sources included windshield tours, store audits, and GIS analyses, all described in detail below.

### 2.1. Windshield tours

Windshield tours were completed by the same person in both Lenoir County and East Los Angeles in order to gain an understanding of food environment context and community conditions. A member of the research team drove to previously identified corner stores, writing detailed qualitative observations and descriptions of community characteristics and context on a field document. The environmental features that were recorded included the presence of sidewalks, the proximity of corner stores to schools, neighborhood areas, graffiti and disrepair, level of isolation, parking lot size, proximity to other food stores, and corner store appearance. Notes were then digitally recorded and organized for theme analysis. The methods used were similar to those used in previous research [64],[65].

### 2.2. Food Store audits

Structured observations, including detailed food store audits, were conducted in a sample of corner stores (Los Angeles: 161; Lenoir: 28) in both counties in order to understand corner store food availability. Different audit tools were used between the two locations, as the audits were done independently as part of separate larger studies. This study utilized the relevant matching observations pertaining to the availability of specific foods, which were comparatively analyzed across the two regions.

The methods used for the store audits in Lenoir County are described elsewhere in greater detail [66]. Briefly, corner stores were identified from the Reference USA business database using the search terms “convenience store” and “grocery store”, corresponding to Standard Industrial Classification (SIC) codes: 541103l and 541105 respectively, and North American Industrial classification Codes (NAICS) 445110 and 445120, respectively. Only grocery stores with fewer than 20 employees were considered corner stores. Stores were selected to be audited based upon multiple factors, including proximity to low income neighborhoods and estimated sales volume (as reported in the Reference USA business database). Audits of North Carolina stores were conducted by trained research assistants using the Nutrition Environment Measures-Stores-Revised (NEMS-S-Rev), a validated tool that measures availability, pricing, and quality of foods at food stores [67]. Additional data was collected on variables of interest including availability of canned foods and presence of hot food grills. In each store, trained research assistants completed the NEMS-S-Rev independently, and then met to discuss and resolve discrepancies between coding decisions so that final inter-rater reliability was 100%.

In Los Angeles County, corner store audits were conducted in five distinct communities in the county, including three predominantly low-income Latino communities (East LA, South LA, and Boyle Heights) and two more upscale, predominantly White communities (Culver City, Manhattan Beach). A list of licensed retail food outlets was identified through the Los Angeles County Environmental Health website [68]. The survey instrument was from an instrument originally adapted by Community Health Councils [69], updated and beta tested by four researchers in 2012. The East LA food retailer survey included assessments of store type, availability, cost, variety and quality of fruits, vegetables, grains, meats, dairy, oils, beverages, and snack foods. From June, 2012-April, 2013 trained undergraduate and graduate nutrition students conducted audits of 161 retail food outlets in these five communities. At least two students visit each store together. When there were questions about the data, the store was either called or data collectors went back to visit in order to confirm or change the data, as appropriate.

When the audits were completed, the corner store data was compiled for analysis, and descriptive statistics were conducted and results generated. Pearson's chi-square (chi2) tests were used to examine differences in food availability between the two settings.

### 2.3. Spatial availability

GIS software (ArcGIS (ESRI, Inc, Redlands, CA) was used to examine the spatial distribution of stores and food products for both Los Angeles and Lenoir Counties. Corner stores and supermarkets were batch geocoded using the Google Maps geocoding Application Programming Interface (API) through the BatchGeo website. Lenoir store locations were verified using satellite imagery and ground truthing. The Los Angeles data were not further verified, but a sample of 300 stores indicated that 80% were geocoded to the rooftop level (the most accurate level), and the rest were range interpolated. Previous research has indicated that using this method is a good, accurate option to geocode addresses [70]. To understand spatial differences in food availability, ArcGIS was used to provide spatial designation of each corner store food attribute value (derived from the food store audits), and then spatial visualization was used to enumerate results.

Corner store locations were spatially linked to the census tract corresponding to the store street address, and then spatial data were joined to an attribute data table with selected variables of interest from the 2010 Decennial Census. Census tracts were categorized into high (> $46,000) and low income ( < $46,000) for both Lenoir and Los Angeles (corresponding to 200% federal poverty level guidelines for a family of 4) [71], average Median Household Income (MHHI), SNAP eligible (average MHHI $31,000 and less) and non-SNAP eligible (average MHHI $31,000 and more), higher and lower percent Hispanic (highest versus lowest quartile), and higher minority(80% or more percent minority; selected because Los Angeles food store audits were in predominantly Hispanic neighborhoods) versus lower Minority (less than 80% percent minority). In order to understand the food environment context around corner stores, the distance from each corner store to the closest supermarket was determined using ArcGIS Network Analyst Closest Facility Analysis.

We evaluated the proportion of stores with fruit and vegetables available for sale in low versus high income areas, SNAP eligible MHHI, higher vs lower Hispanic, and higher versus lower Minority areas using chi2 test for the Los Angeles stores, and Fishers exact test for the Lenoir County stores (more approximate for small sample sizes).

### 2.4. Spatial regression analysis

To understand the differences in predictors of corner store presence (urban versus rural), a spatial regression analysis was performed for 1) Los Angeles County, and 2) a seven county area in rural eastern North Carolina, including Lenoir and six demographically similar surrounding counties (Greene, Onslow, Craven, Duplin, Jones, Wayne).

Given that our dependent variable count data was over-dispersed and the variance exceeded the mean for both the urban and rural study areas, a Negative Binomial regression was conducted to examine correlates of corner store counts within each Census Tract in both areas (Rural: 111 Census Tracts; Urban: 2346 Census Tracts), with the following census tract-level correlates: No Vehicle Households (percent of households without vehicle), Hispanic Population (percent), Minority Population (percent), MHHI, Total Population, and Supermarket count. No Vehicle Households and MHHI were used because it was hypothesized that corner stores may be located near residential areas with lower incomes and limited transportation, places where traditional supermarkets may be less successful. Percent Hispanic and Percent Minority were used given that corner stores or “bodegas” are commonly accessed by these populations [33], and initial findings from this study seemed to indicate a higher corner store presence in census tracts with larger Hispanic populations. Supermarket count was used as it was considered that the presence of larger food stores may influence the presence of smaller food stores. Total population size was used as a variable of interest because the size of the potential customer base may influence the presence of a corner store or supermarket. Multi-collinearity was assessed looking at the variance inflation factor, and was not deemed to be an issue. Negative Binomial Regression was conducted using RStudio.

The data were then assessed for spatial autocorrelation (non-spatially independent observations which could bias results [72]) using the Moran's I measure, applying the Euclidean Distance contiguity approach for polygons. The Moran's I score for the Los Angeles County data was 0.282 (z-zcore 24.70; *p*-value < 0.001), indicating positive statistically significant spatial autocorrelation (positive correlation between the amount of corner stores in a census tract and that of its geographical neighboring census tracts). Given this spatial autocorrelation, the LaGrange statistics were assessed to determine how to incorporate this spatial dependence into the regression model. The Robust LaGrange Multiplier-Lag statistic was statistically significant (LM-Lag = 16.7132; *p*-value = < 0.001), indicating the need for a spatial lag due to spatial dependence among the census tracts, while the Robust LaGrange Multiplier-Error statistic was not statistically significant, suggesting the spatial error model was not appropriate. For the rural county data, the Moran's I (-0.4830; *p* = 0.63 ) was not statistically significant, indicating there was not sufficient autocorrelation to be concerned. The Robust LaGrange Multiplier-Lag statistic was not statistically significant (LM-Lag = 0.0482; *p*-value = 0.83), indicating that a spatial lag was not needed. Spatial Lag Regression (spatially lagged dependent variable; Maximum Likelihood Estimation) was then completed for the urban area data (using the same variables from the Negative Binomial) using a first order queen contiguity (all touching census tracts were neighbors) to determine neighborhood structure in order to assess for similarity amongst the census tracts. The Spatial Lag regression was completed in GeoDa.

We hypothesized that there might be different contextual influences that would lead to variation in the influence of our independent variables on corner store presence over space. These contextual influences could be underlying geographical, structural, or social conditions which are either products of, or are related to, the independent variables of interest. For instance, the prevalence of a racial or ethnic group in a certain area could result in a modified food environment to meet cultural needs, compared to other areas where that group is less prevalent [73]–[75]. Or existing planning ordinances or policy efforts may be influencing the structure of the food environment as it pertains to corner store location. Thus, a Poisson Geographically Weighted Regression (GWR) [76] was used as a local regression technique in addition to the classical “global” regression for the Los Angeles County data to examine for local variation in the influence of the independent variables of interest. Los Angeles County was analyzed using GWR based on a statistically significant Koenker statistic (*p* < 0.05) indicating data non-stationarity (explanatory variables in the model have an inconsistent relationship to the dependent variable over geographical space). The Rural Counties data did not have a statistically significant Koenker statistic (*p* = 0.08) [77]. The aim was to enhance our understanding of the influence of the variables of interest over space, which might help inform future policy and planning decisions. The variables of interest from the local regression approaches were examined for non-stationarity. An adaptive bi-square kernel was used to clarify local extents for model fitting. The bandwidth was set at optimal values (Limits 54, 2346), using the golden-section search option in GWR4. ArcGIS version 10.1 and GWR4 were used for GWR analysis.

## 3. Results

### 3.1. Windshield tours

Los Angeles County corner stores were often located within neighborhood areas and in close proximity to schools, with school children frequently seen shopping at the stores. Lenoir County stores were often more isolated, less accessible, but having larger stores and parking lots, and more free space surrounding the store compared to the Los Angeles urban stores. Graffiti and disrepair affecting the appearance of stores was more common in the urban areas compared to the rural areas, including spray paint “tagging” and barred windows for security. In both rural and urban locations, junk food and junk food advertisements were prominently displayed at store entrances.

### 3.2. Food store audits

Culturally specific junk foods, including pastries (i.e. pan dulce, mini pies, etc.), chips, and snack foods (i.e. spicy corn chips, beef jerky, etc.), were found in both locations. Chips and snack foods often used marketing strategies that targeted particular demographic groups for each location, including spicy chips with Hispanic-oriented labeling in Los Angeles, and “southern” or “southern style” labeled foods in Lenoir County.

Los Angeles stores were more likely than Lenoir County stores to have fresh fruits (112/161 (69%) versus 5/28 (18%); chi2 = 27.04; *p* < 0.001), and vegetables (112/161 (70%) versus 5/28 (18%); chi2 = 27.04; *p* < 0.001), though Lenoir stores did often have canned fruits (6/28 = 21%) and vegetables (13/28 = 46%) available. There was a non-significant (chi2: 3.1663; *p* = 0.075) trend for rural stores to be more likely to have grills offering prepared food (7/29; 24%) compared to urban stores (19/161; 12%).

### 3.3. Spatial availability

In Los Angeles, corner stores in the highest income quartile (11/13 = 84%) were not significantly more likely to have fruits compared to the lowest income quartile (79/114 = 69%) (chi2 = 1.321; p-value = 0.25). In Lenoir, there was no difference (*p* = 1.0) in corner stores in the highest income quartile (0/3 = 0%) compared to the lowest income quartile (1/6 = 16%). In Los Angeles, corner stores in the highest income quartile (4/13 = 31%) were significantly less likely to have vegetables compared to the lowest income quartile (88/114 = 77%) (chi2 = 12.59; p-value = 0.001). In Los Angeles, corner stores in census tracts with MHHI above SNAP benefit level of $31,000 were not significantly more likely to have fresh fruit (93/132 = 70% versus 19/29 = 66%; chi2 = 0.27; *p* = 0.60) compared to those below the SNAP-EBT threshold, while in Lenoir County, corner stores above and below the SNAP threshold (2/15 = 13% versus 3/10 = 30%; *p* = 0.36) did not significantly differ in fresh fruit availability. In Los Angeles, corner stores in areas above SNAP benefit level of MHHI of $31,000 were not significantly different in fresh vegetable availability (88/132 = 67% versus 23/29 = 79%; chi2 = 1.78; *p* = 0.18), while in Lenoir County, corner stores in above and below SNAP level MHHI areas (1/15 = 16% versus 1/10 = 10%; *p* = 1.0) did not differ in fresh vegetable availability.

In Los Angeles, corner stores in the highest quartile of percent Hispanic were more likely (chi2 = 16.16; *p* = 0.002) to have fresh vegetables (93/137 = 76%) compared to areas of low Hispanic population (3/13 = 23%), whereas there was a low presence of stores with fresh vegetables available in the highest quartile of percent Hispanic in Lenoir (0/9 = 0%), though this difference was not significantly different than areas with lower Hispanic populations in Lenoir (0/5 = 14%) (*p* = 0.49). In Los Angeles, corner stores in the highest quartile of percent Hispanic were not more likely (chi2 = 0.36; *p* = 0.54) to have fresh fruit (104/137 = 75%) compared to the lowest quartile of Hispanic population (6/24 = 25%), and no difference in fresh fruits available in the highest percent Hispanic quartile Lenoir (1/9 = 11%), though this difference was not significantly different areas with lower Hispanic populations in Lenoir (0/5 = 0%) (*p* = 1.0).

Fresh vegetable availability was not significantly different in Lenoir Higher Minority versus Lesser Minority areas (1/6 = 16% versus 1/19 = 0.05) (*p* = 0.43), but was significantly greater in Higher Minority (107/141 = 76%) areas of Los Angeles compared to Lesser Minority (5/20 = 25%) areas (chi2 = 21.4223; *p* = .001). In Los Angeles, there was no significant difference in fresh fruit availability in Lower Minority (20% of Census Tract) (5/20 = 75%) versus Higher Minority (46/142 = 32%) areas (chi2 = 0.44; *p* = 0.51), as well as in Lenoir High Minority versus Low Minority: 1/6 = 16% versus 4/19 = 21%) (*p* = 1.00).

The average distance between corner stores and supermarkets in Lenoir County was 1.6 (SD = 1.65) miles, with those corner stores outside of the county seat, in the most rural areas, (25/70, 36%) being an average of 3.1 miles (SD = 1.88) from the nearest supermarket (furthest distance being 7 miles), and with those corner stores inside the city limits (45/70 = 64%) being an average of 0.73 miles (SD = 0.45) from the nearest supermarket (furthest distance being 1.74 miles). The average distance between corner stores and regional supermarkets in LA was 1.2 miles (SD = 0.66), with the farthest distance being 2.6 miles. There was not a statistically significant difference (using a t-test) in overall distance between the rural and urban area (*p* = 0.06).

### 3.4. Spatial regression

For Los Angeles County, Spatial Lag regression (see Table 2) indicated that the predictors: total population, no transportation and Hispanic population were positively associated (*p* < 0.05) with corner store count, indicating increased corner store presence in locations with greater total populations, No Transportation and Hispanic population. Median Household Income (*p* < 0.001) and Minority Percent (*p* < 0.05) was inversely associated with corner store count, indicating a lower corner store presence in locations with greater household incomes. Supermarket count was not significantly associated with corner store count (*p* > 0.05). A test of non-stationarity (Koenker statistic) was significant (*p* < 0.05). The overall R-squared (pseudo) measure of model fit was R-squared = 0.26. The lag coefficient (Rho) was statistically significant (rho: 0.38; *p* < 0.001), which further suggests spatial dependency (the propensity for nearby locations to influence each other and to possess similar attributes [78]) in the data. This suggests improved model fit using the spatially lagged dependent variable compared to a regression method that did not incorporate this spatial dependence (in our case Negative Binomial), which would have biased the results [72].

**Figure 1.**
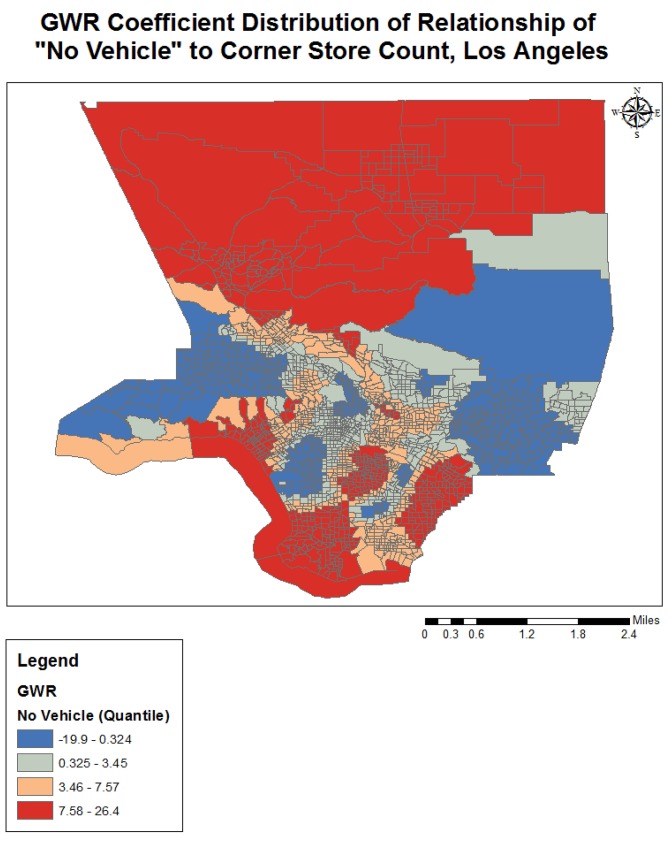
GWR Coefficient Distribution of Relationship for No Vehicle to Corner Store Count, Los Angeles

**Figure 2.**
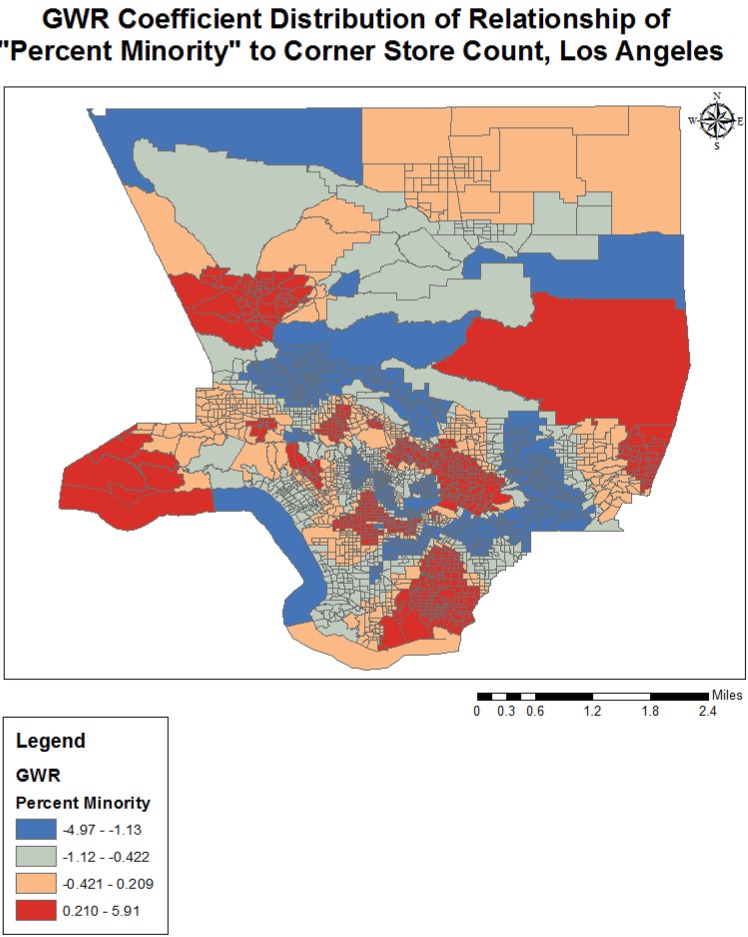
GWR Coefficient Distribution of Relationship for Percent Minority to Corner Store Count, Los Angeles

**Figure 3.**
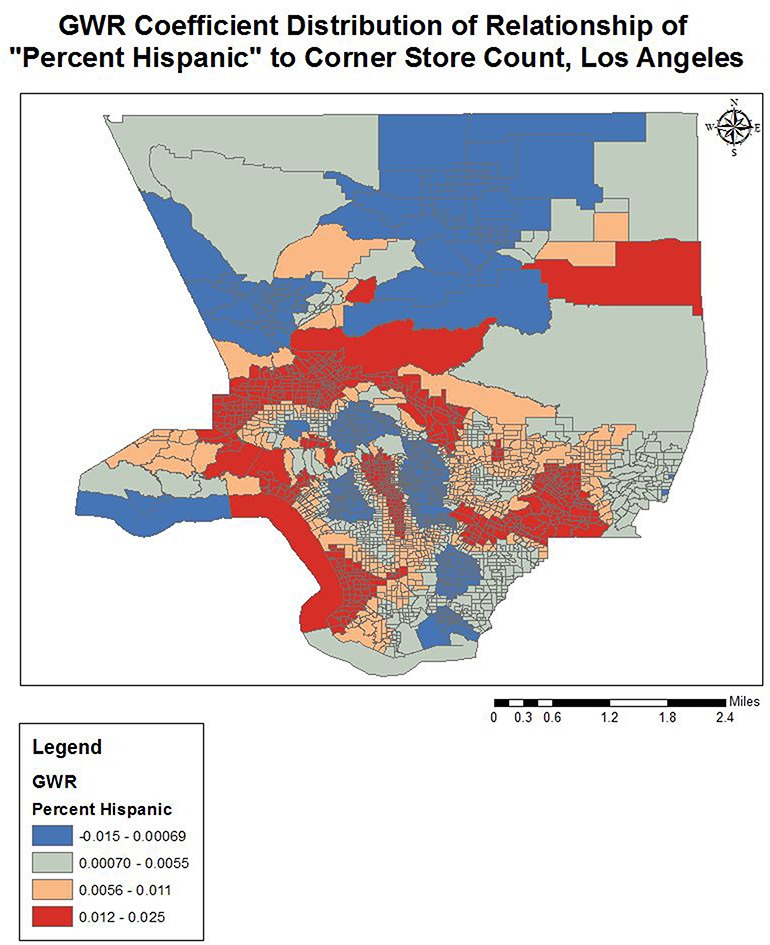
GWR Coefficient Distribution of Relationship for Percent Hispanic to Corner Store Count, Los Angeles

**Figure 4.**
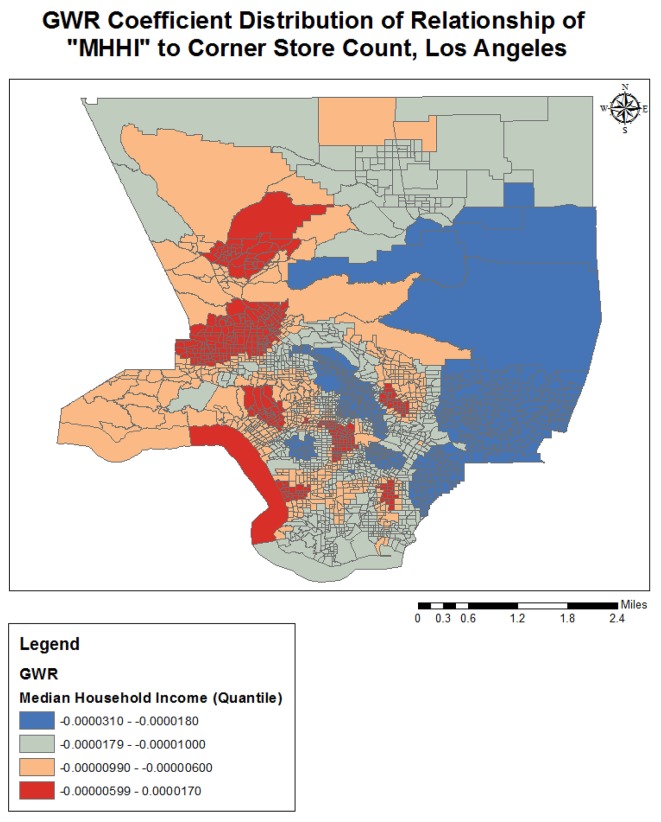
GWR Coefficient Distribution of Relationship for MHHI to Corner Store Count, Los Angeles

**Figure 5.**
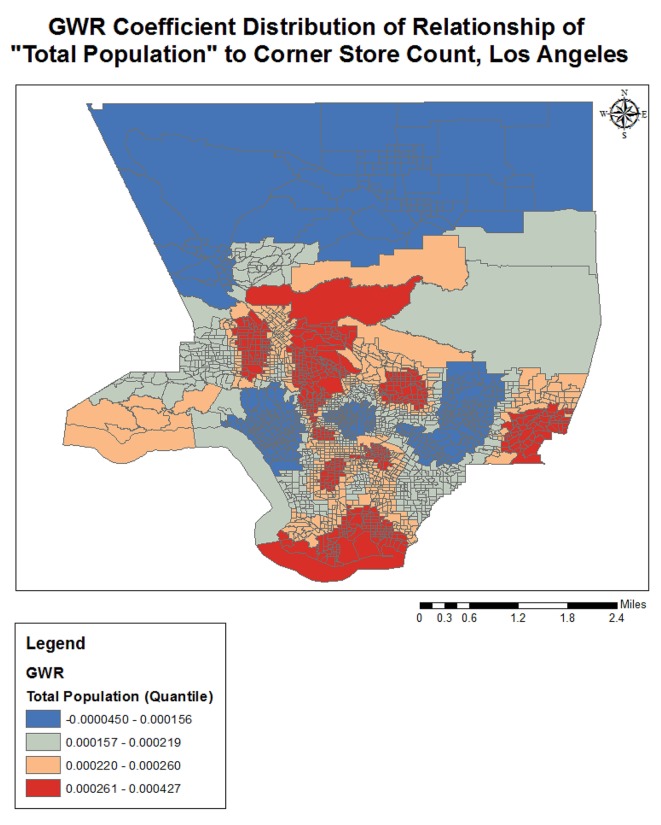
GWR Coefficient Distribution of Relationship for Total Population to Corner Store Count, Los Angeles

Completing a GWR improved the overall model fit (R-squared went from 0.25 to 0.38), and performance (AICc decreased from 3687.2 to 3298.1) further indicating variation in the relationships between the predictors and outcome variable over geographical space (see Table 3). The quantile classification scheme was used to assess model variation over space, and data maps of relevant variables (population density, income, racial dominance, etc.) were referenced. The variables all showed significant variation over space (see Figures 1–5). The No Vehicle variable was most positively associated with corner store count in the high Hispanic East LA area, West LA (university area), and less dense northern county area, but negatively associated in the Metro and lower San Fernando Valley area. The Minority Percent variable had a positive relationship in higher Hispanic areas like the San Fernando Valley area and Eastern Los Angeles, as well as the predominately Black areas, but a strongly negative association in White and Asian/Pacific Islander dominated areas. The Hispanic Percent variable had a positive relationship with corner stores in certain high Hispanic areas of town (East and Central LA) and less in others (San Fernando Valley). For the Median Household Income variable, the model showed a stronger inverse association in west and northern LA County, and a weaker inverse association in South and East LA County. For the Total population variable, variation seemed to indicate a positive relationship with corner store count in lower income areas, and a negative relationship in the more affluent areas. The overall model had the best fit (based on the local R-squared values) in southern and eastern LA County (generally lower income), and the worst fit in the West and Central LA, indicating other variables may be influencing corner store count in those areas.

**Table 2.** Los Angeles County, Regression results (Negative Binomial and Spatial Lag) showing the association between Corner Store Count and No Vehicle Households (Percent), Hispanic Percent, Median Household Income, Total Population, and Supermarket Count.

Negative Binomial			
Variable	Coefficient	Sd. Error	Probability
No Vehicle Households (Percent)	3.177	5.320e-01	< 0.001*
Hispanic Percent	2.983e-03	1.151e-03	0.009*
Minority (Percent)	-1.372e-01	1.462e-01	0.32
Median Household Income	-1.489e-05	1.191e-06	< 0.001*
Total Population	1.853e-04	1.393e-05	< 0.001*
Supermarket Count	1.012e-01	6.112e-02	0.09

Spatial Lag			
Variable	Coefficient	Sd. Error	Probability
CS Count (lag)	0.30	0.03	< 0.001*
No Vehicle Households (Percent)	2.76	0.44	< 0.001*
Hispanic Percent	0.003	0.007	< 0.001*
Minority (Percent)	-0.18	0.08	0.04*
Median Household Income	-5.61e-006	6.24e-007	< 0.001*
Total Population	0.0001	9.53e-006	< 0.001*
Supermarket Count	0.11	0.10	0.26

*Statistically significant at 0.05

**Figure 6.**
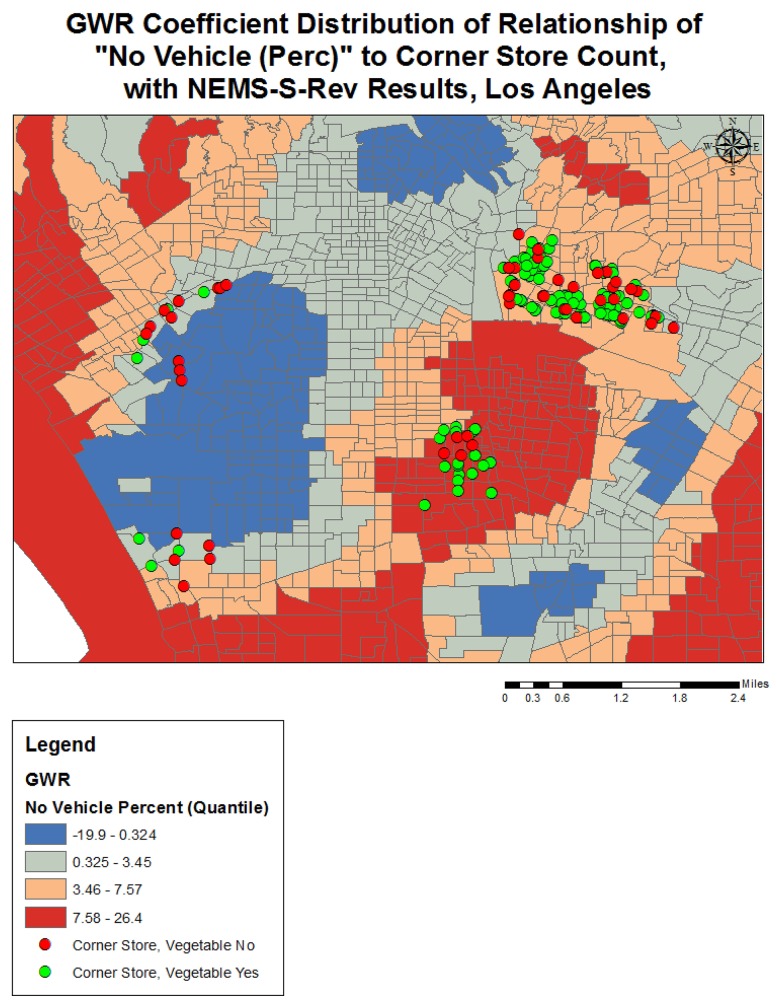
GWR Coefficient Distribution of Relationship for Total Population to Corner Store Count, with NEMS-S-Rev Results, Los Angeles

In examining the visual relationship between NEMS food store audit findings and the GWR results, most of the visualization suggested no clear possible differences, except for the No Vehicle variable (see Figure 6). There appeared to be a greater presence of vegetables available where the relationship between No Vehicle percent and Corner Store Count was positive compared to where it was negative, indicating that in places with limited transportation and high corner store count, there may be greater availability of fresh vegetables. This difference was not seen with fresh fruit.

**Table 3.** LA County GWR Results.

GWR Results					
**Bandwidth Size**	201.2				
**Deviance**	2880.9				
**AICc**	3298.1				
**Percent Deviance** **Explained**	0.38				

Geographically varying (Local) coefficients					
Variable	Mean	STD	Min	Max	Range
Intercept	0.11	1.33	-6.19	5.19	11.38
No Vehicle Households (Perc)	4.050	6.97	-19.91	26.35	46.26
HISPANIC (Perc.)	0.005	0.007	-0.02	0.03	0.04
MHHI	-0.000012	0.000008	-0.00003	0.00002	0.00005
Total Population	0.0002	0.000080	-0.00005	0.0004	0.0005
Minority (Perc.)	-0.40	1.24	-4.98	5.91	10.88
SM Count	-0.007	0.35	-1.37	0.99	2.35

In Rural Eastern NC (including Lenoir), negative binomial regression (see Table 4) indicated that Total Population (*p* < 0.05) and Supermarket Count were positively associated (*p* < 0.001) with corner store count, indicating increased corner store presence in locations with greater total population and more supermarkets. Median Household Income was significantly negatively associated with corner store count (*p* < 0.001), indicating a higher corner store presence in locations with lower household incomes (and more households with no vehicles). The variables No Transportation, Percent Hispanic, and Minority Percent were not significantly associated with corner store count. There was not a statistically significant non-stationarity effect (Koenker (BP) Statistic (*p* > 0.05)).

**Table 4.** Lenoir County and surrounding area (Rural Eastern NC), Spatial Regression Results showing the association between counts of Corner Stores and No Vehicle Households (Percent), Hispanic Percent, Median Household Income, Total Population, and Supermarket Count.

Variable	Coefficient	Sd. Error	Probability
No Vehicle Households	-6.72e-01	4.06	0.86
Hispanic Percent	-6.79e-03	8.59e-03	0.43
Median Household Income	-1.63e-05	5.96e-06	0.006*
Total Population	8.59e-05	2.854e-05	0.003*
Supermarket Count	1.94e-01	5.29e-02	< 0.001*
Minority Percent	6.96e-03	3.68e-03	0.06

*Statistically significant at 0.05

## 4. Discussion

Spatial differences in the availability of healthy food products in corner stores suggest that offerings may vary by location, and are influenced by a number of important store and contextual attributes (e.g. ethnic composition and store isolation). Our findings indicate important contextual similarities and differences between corner stores in urban and rural locations. Interestingly, the presence of a Hispanic population was positively associated with corner store count in the urban area, but not in the rural area. This may be due to differences in cultural dominance and establishment in the two locations under study. Previous research has shown that Hispanics prefer to shop at smaller, more personal, ethnic stores [79]. If the Hispanic culture is more dominant and established in an area, this influence could be reflected by a proximal food environment geared towards those preferences, whereas an area with less Hispanic influence would match the preferences of the dominant cultural group. More work should be done to understand whether this occurrence is widespread, and if so, to understand underlying causes of these differences. There was also a positive association with Percent Minority, which was close to being statistically significant in both locations, but just above the significant threshold. The results for both the Hispanic and overall Minority data may not be simply related to race or ethnicity, but are likely tied into larger social and cultural structures at play which include racism, opportunity, social interaction, and cultural values [73]–[75]. The presence of corner stores could be a sign of one or many of these structures.

In both places, Median Household Income was a driving factor in corner store count, indicating a consistent link regardless of residential density, with a higher income associated with fewer stores. This may be due to higher income households having their needs met by nearby supermarkets and therefore unlikely to need something from a smaller corner store. Alternatively, it suggests a greater amount of corner stores in lower income areas, which is consistent with previous literature. Sharma (2014) [57] suggested that spatially evaluating socio-economic variables are important to health promotion research, as they are indicators of spatial distribution of larger constructs such as knowledge, power, and social connections [73]. This information can be used to understand the ability for residents of a geographic area to maintain a healthy lifestyle, and support positive, health promoting change in their surroundings [73]. Our findings may indicate that the ability of residents in low income areas to effectively shape their food environment to promote healthy living is diminished compared to their higher income counterparts, leading to small food stores with typically less healthy offerings. This could be the result of multiple factors, including a widespread lack of overall knowledge of healthy eating which diminishes demand for healthier items, or through inopportunity to establish health food stores through lack of power and social and monetary capital. Determining the driving forces behind these food access issues is critical to enacting sustainable change, and should be considered when attempting to do any corner store transformation work in lower income areas.

There were also differences in the association of corner store count with households not owning any vehicles, with the variable of “No Household Vehicles” being inversely associated with counts in high income areas, and positively associated with counts in low-income areas. Stores in urban areas may be located in areas of high urban density, where household vehicle ownership is less required for access to amenities needed for daily living than in rural areas. They also offer residents more proximal food access compared to larger food stores such as supermarkets.

The statistically significant lag coefficient for the urban area suggests spatial dependency, an issue where corner store count in one place is affected by the independent variables in other places. Spatial dependence could indicate that there is measurement error in using administrative boundaries that do not accurately reflect the nature of the underlying processes generating the sample data, or more importantly could be indicative of the impact of social and economic processes across space [72]. Our results are suggestive of a possible diffusion process, where events in one place predict an increased likelihood of events in neighboring places [72]. Interestingly, this spatial dependency was not present in the more rural area (though this could be due to the measurement error of the use of administrative boundaries previously mentioned). The more isolated nature of rural corner stores may inhibit the cultural and structural diffusion processes leading to corner store presence which appears to be happening in the urban area. This should be explored further to help us understand the type and nature of the diffusion process that is occurring, as it may help improve our understanding of the location of corner stores given certain market conditions.

The fact that there was data non-stationarity in factors associated with corner store count in the urban area but not in the rural area is also an interesting finding. This suggests that in urban areas there are more local influences associated with corner store count, and in rural areas there appears to be more large scale, consistent relationships between the examined variables and corner store count. This may have important implications for food environment planning and policy, as these varying influences should be considered.

This idea of meeting a proximal food need in a “food desert” was supported by the fact that in Los Angeles, corner store count was not associated with supermarket presence. This theme appeared to not hold for rural areas, where the expectation would be that many corner stores would be located in crossroads areas to meet rural food needs. Corner stores may be foregoing these isolated crossroads given greater customer traffic surrounding supermarket shopping centers, and given the already high household ownership of vehicles to meet daily transportation needs in rural areas. Urban corner stores are distinctive for being pedestrian-friendly whereas local supermarkets are car-friendly [80]. In rural areas, all stores, big and small, may need to be car-friendly because of the longer distances between residences and stores. This result could have also been influenced by the inclusion of corner stores with gas stations, which tend to cluster around densely populated shopping areas. Interestingly, this idea is not supported by our findings that the distance between corner stores and supermarkets in rural areas was greater than in urban areas, which was expected. Given the discrepant findings, this issue should be studied further.

The Geographically Weighted Regression analysis involving Los Angeles County seemed to indicate differences in the relationship between racial domination and corner store count, with higher Hispanic and Black areas having a positive relationship with corner store counts, and areas with predominately Whites and Asians having a negative relationship. There was also some fluidity in the relationship between Hispanic status and corner store count over space. This may be due to income differences between areas, as suggested by the corresponding maps of median household income. In this large urban setting, the likely presence of urban density, business density, and public transportation services appeared to influence the direction of the relationship between vehicle availability and corner store count, as the relationship was reversed from positive to negative in these areas. This suggests a confluence of multiple factors leading to corner store presence. The indication that there may be greater availability of fresh vegetables in places where there is limited transportation and high corner store count is an interesting finding, and should be further examined in a larger sample. Overall, these statistically significant variables exhibited strong regional variation, which suggests that local policy, more so than large regional policy, can be informed by this information.

Our findings are comparable to findings in previous related research. Morris, Neuhauser, and Campbell (1992) [81], similarly found a low availability of produce in smaller food stores in rural areas, though they did not compare to stores in urban areas. We found that produce was more available in the urban compared to rural stores, though junk food was common to both. This is suggestive of a need to improve the accessibility of fresh produce in rural stores, and reduce the amount of unhealthy foods in food stores in both places. Interestingly, we also found that fresh vegetables were more available in higher versus lower minority areas of the urban area of Los Angeles, where the study area was largely Latino. Grigsby-Toussaint et al (2010) [82] did find a greater amount of fresh produce in urban corner stores in Latino versus African American neighborhoods, but they did not compare availability with corner stores in predominantly white neighborhoods. This, along with our findings, may indicate a unique Hispanic cultural influence on the availability of fresh produce in small corner stores, which should be further explored.

Powell et al (2007) [10] found a greater density of food stores in urban US zip codes, similar to our results, though they did not directly compare distances between food outlet types. They also found that convenience stores were more common in lower income zip codes, a finding similar to what we found at the census tract level (a more precise geographic area). This is also similar to findings from Sharkey et al in rural Texas [83],[84], which found that convenience stores were associated with area deprivation (which included household income). Our findings differed from their findings in regards to a positive association with no vehicle households, where our study found a positive association in the urban but not the rural area. Differences could be due to that fact that the rural Texas study area was both more populated and had greater population density than the North Carolina study area [84]. The Sharkey et al research [84] was conducted in a largely Hispanic area (88%), with similar findings to what our study found in a largely Hispanic urban LA area. Our research, along with the Sharkey research, appears to be of the few studies to examine the availability of corner stores in relation to Hispanic populations in a rural setting. As the Hispanic population in rural areas grows, more research is needed to better understand this relationship.

Other research by Morland et al (2002) [34] found that convenience stores with gas stations were more likely in higher income census tracts, but convenience stores without gas stations were more common in low income neighborhoods. They also found that convenience stores with gas stations were more prevalent in mixed neighborhoods than in predominantly black neighborhoods. Our findings were similar to this, as Minority Percent was not statistically significant (though close). Galvez et al (2008) [33] found that convenience stores were more likely to be located in predominantly Hispanic neighborhoods and less likely to be located in Black neighborhoods in New York City, a result similar to what we found in our examination of urban Los Angeles. Lee et al found that convenience stores were more prevalent in low income and high minority areas across two urban locations, which matches our low income findings but not completely with our minority findings [85]. These inconsistences should be further explored.

In rural North Carolina, we found a much greater distance from corner stores to supermarkets. This may suggest that corner stores play a more pivotal role in improving access to food sources in rural areas. Thus, there should be increased emphasis on making these food stores a source of healthy foods for the surrounding populations, who otherwise would have limited access.

In both places, promoting healthier culturally specific foods, and discouraging culturally specific junk foods, may be an effective intervention approach. Prominent displays of culturally advertised junk foods were seen in both areas, mostly displayed on aisle endcaps, at the front entrance, and at the cash registers. This is termed “Culturally Target Marketing”, where the goal is to get certain groups to purchase or consume more of an item [86], in this case, junk food. Cultural targeting of junk foods is a public health concern [87], as it encourages the consumption of foods that people should limit, perpetuates cultural stereotypes, and exploits cultural identities [88]. Thus replacement of these items with healthier culturally specific foods may make the healthier choice the easier choice for store patrons and promote healthier cultural practices, while also promoting high profit margin items like fruits and vegetables [89].

Rural stores have additional opportunities for transformation given their larger lot and store size. These attributes are important as they provide the opportunity for activities like healthy food production and storage of produce and necessary refrigeration units. Healthy food production through the small gardening of available open space may be a cost efficient and profit maximizing approach to increase the availability of produce in stock at the corner store. Also, given the more frequent availability of prepared foods and grills in rural corner stores, there are opportunities for additional transformation, including recipes and offering healthy modifications of standard menu items.

Limitations of the study include the cross sectional nature of the research, the possible limits on the representativeness of this data and issues with generalizability beyond the study settings, potentially subjective findings derived from the qualitative windshield tour method, and measurement error associated with identifying food outlets from a commercial business database that may not reflect all of the recent food store openings and closings [90]. Another limitation was the limited number of food store audits in Lenoir County, and the fact that the LA food store audits were clustered in high Hispanic areas, limiting analysis. Also we did not have the resources to examine differences in rural and urban areas within the same state, which would have been informative. However, the approach in this paper contrasting such starkly different contexts will likely be more informative to future intervention translation efforts. While efforts were made to reduce error from inaccurate geocoding through vetted methodology, some geocoding-related error may still exist. Using census tracts, which are point based measures aggregated into potentially arbitrary districts, and which create issues like edge effects, can introduce error and statistical bias into the results [91]. The fact that audits were conducted in the two areas at slightly different time points could be a potential limitation, though large variation in the overall presence of fruits and vegetables was not expected to change by season compared to particular fruit and vegetables types. The use of place-based metrics in this study may be limited by the assumption that people in a particular location have the same level of food accessibility, even though other factors like personal context and time budgetary constraints may be influential. These results should be paired with future consumer based metrics, as suggested by Horner and Wood (2014) [58]. They suggest that offering a fusion of place based metrics, the focus of this paper, along with more people-based metrics of individual travel context, may lead to more valuable insights into real food accessibility, which would help clarify what policy change or intervention would be most effective [58]. Thus, future research should incorporate individual level metrics including personal time availability, mode of transportation, personal shopping preferences, and travel routes, which would help understand relative food opportunities and interaction with corner stores along individual paths for rural and urban consumers. Strengths of this study include the use of a mixed-method, multifaceted research approach, conducting detailed food store audits to determine actual versus assumed food exposure, and using spatial regression techniques to take into account such factors as spatial autocorrelation, and examining spatial non-stationarity.

## 5. Conclusion

Our study indicates important similarities and differences between corner stores in urban and rural environments. In both settings, corner stores provide an important point of access to food, but provide different food types, have distinct roles in improving food access in their respective locations, and meet distinct cultural needs for their particular contextual settings. Our findings suggest that acknowledgement of these similarities and differences may be critical to understanding the potential translational success of corner store transformation efforts between these two settings, and that blanket policy may not be nuanced enough to promote effective change in each environment. Thus, a more targeted approach is likely needed in each setting to maximize program effectiveness and impact. Geographic variation in the relationships between variables of interest and corner store count suggests that local contextual and spatial factors may influence corner store location and the food products stocked by the store. The distribution of corner stores is important to understanding the food landscape and has important implications for planning efforts to improve the food environment. The spatial location of corner stores, as well as the micro food environment within those stores, should be further studied to improve understanding of the role that corner stores can play in optimizing communities' access to healthy foods in both urban and rural settings.
